# Inferior Frontal Sulcal Hyperintensities on Brain MRI Are Associated with Amyloid Positivity beyond Age—Results from the Multicentre Observational DELCODE Study

**DOI:** 10.3390/diagnostics14090940

**Published:** 2024-04-30

**Authors:** Marc Dörner, Katharina Seebach, Michael T. Heneka, Inga Menze, Roland von Känel, Sebastian Euler, Frank Schreiber, Philipp Arndt, Katja Neumann, Annkatrin Hildebrand, Anna-Charlotte John, Anthony Tyndall, Johannes Kirchebner, Pawel Tacik, Robin Jansen, Alexander Grimm, Solveig Henneicke, Valentina Perosa, Sven G. Meuth, Oliver Peters, Julian Hellmann-Regen, Lukas Preis, Josef Priller, Eike Jakob Spruth, Anja Schneider, Klaus Fliessbach, Jens Wiltfang, Frank Jessen, Ayda Rostamzadeh, Wenzel Glanz, Jan Ben Schulze, Sarah Lavinia Florence Schiebler, Katharina Buerger, Daniel Janowitz, Robert Perneczky, Boris-Stephan Rauchmann, Stefan Teipel, Ingo Kilimann, Christoph Laske, Matthias H. Munk, Annika Spottke, Nina Roy-Kluth, Michael Wagner, Ingo Frommann, Falk Lüsebrink, Peter Dechent, Stefan Hetzer, Klaus Scheffler, Luca Kleineidam, Melina Stark, Matthias Schmid, Ersin Ersözlü, Frederic Brosseron, Michael Ewers, Björn H. Schott, Emrah Düzel, Gabriel Ziegler, Hendrik Mattern, Stefanie Schreiber, Jose Bernal

**Affiliations:** 1 German Center for Neurodegenerative Diseases (DZNE) within the Helmholtz Association, 39120 Magdeburg, Germany; katharina.seebach@gmx.de (K.S.); inga.menze@dzne.de (I.M.); frank.schreiber@dzne.de (F.S.); philipp.ulbrich@dzne.de (P.A.); solveig.henneicke@med.ovgu.de (S.H.); wenzel.glanz@dzne.de (W.G.); falk.luesebrink@dzne.de (F.L.); emrah.duezel@dzne.de (E.D.); gabriel.ziegler@dzne.de (G.Z.); hendrik.mattern@ovgu.de (H.M.); stefanie.schreiber@med.ovgu.de (S.S.); jose.bernalmoyano@dzne.de (J.B.); 2 Department of Consultation-Liaison-Psychiatry and Psychosomatic Medicine, University Hospital Zurich, University of Zurich, 8091 Zurich, Switzerland; roland.vonkaenel@usz.ch (R.v.K.); sebastian.euler@usz.ch (S.E.); jan.schulze@usz.ch (J.B.S.); sarah.schiebler@usz.ch (S.L.F.S.); 3 Luxembourg Centre for Systems Biomedicine (LCSB), University of Luxembourg, 4367 Belvaux, Luxembourg; michael.heneka@uni.lu; 4 Institute of Cognitive Neurology and Dementia Research, Otto-von-Guericke University, 39120 Magdeburg, Germany; 5 Department of Neurology, Otto-von-Guericke University, 39120 Magdeburg, Germany; katja.neumann@med.ovgu.de (K.N.); annkatrin.hildebrand@st.ovgu.de (A.H.); anna-charlotte.john@st.ovgu.de (A.-C.J.); 6 Department of Neuroradiology, Clinical Neuroscience Center, University Hospital Zurich, University of Zurich, 8091 Zurich, Switzerland; anthony.devere-tyndall@usz.ch; 7 Department of Forensic Psychiatry, University Hospital of Psychiatry Zurich, University of Zurich, 8032 Zurich, Switzerland; johannes.kirchebner@pukzh.ch; 8 Department of Parkinson’s Disease, Sleep and Movement Disorders, University Hospital Bonn, 53127 Bonn, Germany; pawel.tacik@ukbonn.de; 9 German Center for Neurodegenerative Diseases (DZNE) within the Helmholtz Association, 53127 Bonn, Germany; anja.schneider@dzne.de (A.S.); klaus.fliessbach@ukbonn.de (K.F.); frank.jessen@uk-koeln.de (F.J.); annika.spottke@dzne.de (A.S.); nina.roy-kluth@dzne.de (N.R.-K.); michael.wagner@dzne.de (M.W.); ingo.frommann@dzne.de (I.F.); luca.kleineidam@ukbonn.de (L.K.); melina.stark@dzne.de (M.S.); matthias.c.schmid@uni-bonn.de (M.S.); frederic.brosseron@dzne.de (F.B.); 10 Department of Neurology, Heinrich Heine University, 40225 Düsseldorf, Germany; robin.jansen@med.uni-duesseldorf.de (R.J.); svenguenther.meuth@med.uni-duesseldorf.de (S.G.M.); 11 Center for Neurology, Tuebingen University Hospital and Hertie-Institute for Clinical Brain Research, Eberhard Karls University Tuebingen, 72076 Tuebingen, Germany; alexander.grimm@med.uni-tuebingen.de; 12 J. Philip Kistler Stroke Research Center, Massachusetts General Hospital, Boston, MA 02114, USA; vperosa@mgh.harvard.edu; 13 German Center for Neurodegenerative Diseases (DZNE) within the Helmholtz Association, 10117 Berlin, Germany; oliver.peters@charite.de (O.P.); julian.hellmann@charite.de (J.H.-R.); josef.priller@charite.de (J.P.); eike.spruth@charite.de (E.J.S.); ersin.ersoezlue@charite.de (E.E.); 14 Institute of Psychiatry and Psychotherapy, Charité—Universitätsmedizin Berlin, Corporate Member of Freie Universität Berlin and Humboldt-Universität zu Berlin, 14129 Berlin, Germany; lukas.preis@charite.de; 15 Department of Psychiatry and Neurosciences, Campus Benjamin Franklin, Charité—Universitätsmedizin Berlin, 12203 Berlin, Germany; 16 German Center for Mental Health (DZPG), Partner Site Berlin, 10785 Berlin, Germany; 17 Department of Psychiatry and Psychotherapy, Charité—Universitätsmedizin Berlin, 10117 Berlin, Germany; 18 Department of Psychiatry and Psychotherapy, School of Medicine, Technical University Munich, 81675 Munich, Germany; 19 UK Dementia Research Institute (UK DRI), University of Edinburgh, Edinburgh EH16 4SB, UK; 20 Department of Cognitive Disorders and Old Age Psychiatry, University Hospital Bonn, 53127 Bonn, Germany; 21 German Center for Neurodegenerative Diseases (DZNE) within the Helmholtz Association, 37075 Goettingen, Germany; jens.wiltfang@med.uni-goettingen.de (J.W.); bjoernhendrik.schott@med.uni-goettingen.de (B.H.S.); 22 Department of Psychiatry and Psychotherapy, University Medical Center Goettingen, University of Goettingen, 37075 Goettingen, Germany; 23 Neurosciences and Signaling Group, Institute of Biomedicine (iBiMED), Department of Medical Sciences, University of Aveiro, 3810-193 Aveiro, Portugal; 24 Department of Psychiatry, Medical Faculty, University of Cologne, 50924 Cologne, Germany; ayda.rostamzadeh@uk-koeln.de; 25 Excellence Cluster on Cellular Stress Responses in Aging-Associated Diseases (CECAD), University of Cologne, 50931 Cologne, Germany; 26 German Center for Neurodegenerative Diseases (DZNE) within the Helmholtz Association, 81377 Munich, Germany; katharina.buerger@med.uni-muenchen.de (K.B.); robert.perneczky@med.uni-muenchen.de (R.P.); michael.ewers@med.uni-muenchen.de (M.E.); 27 Institute of Stroke and Dementia Research (ISD), University Hospital, LMU Munich, 81377 Munich, Germany; daniel.janowitz@med.uni-muenchen.de; 28 Department of Psychiatry and Psychotherapy, University Hospital, LMU Munich, 81377 Munich, Germany; boris.rauchmann@med.uni-muenchen.de; 29 Munich Cluster for Systems Neurology (SyNergy) Munich, 81377 Munich, Germany; 30 Ageing Epidemiology Research Unit (AGE), School of Public Health, Imperial College London, London SW7 2AZ, UK; 31 Sheffield Institute for Translational Neuroscience (SITraN), University of Sheffield, Sheffield S10 2HQ, UK; 32 Department of Neuroradiology, University Hospital, LMU Munich, 81377 Munich, Germany; 33 German Center for Neurodegenerative Diseases (DZNE) within the Helmholtz Association, 18147 Rostock, Germany; stefan.teipel@med.uni-rostock.de (S.T.); ingo.kilimann@dzne.de (I.K.); 34 Department of Psychosomatic Medicine, Rostock University Medical Center, 18147 Rostock, Germany; 35 German Center for Neurodegenerative Diseases (DZNE) within the Helmholtz Association, 72076 Tuebingen, Germany; christoph.laske@dzne.de (C.L.); matthias.munk@med.uni-tuebingen.de (M.H.M.); 36 Section for Dementia Research, Department of Psychiatry and Psychotherapy, Hertie Institute for Clinical Brain Research, University of Tuebingen, 72076 Tuebingen, Germany; 37 Department of Psychiatry and Psychotherapy, University of Tuebingen, 72076 Tuebingen, Germany; 38 Department of Neurology, University of Bonn, 53127 Bonn, Germany; 39 MR-Research in Neurosciences, Department of Cognitive Neurology, Georg-August-University Goettingen, 37073 Gottingen, Germany; peter.dechent@med.uni-goettingen.de; 40 Berlin Center for Advanced Neuroimaging, Charité—Universitätsmedizin Berlin, 14129 Berlin, Germany; stefan.hetzer@charite.de; 41 Department for Biomedical Magnetic Resonance, University of Tuebingen, 72076 Tuebingen, Germany; klaus.scheffler@med.uni-tuebingen.de; 42 Institute for Medical Biometry, University Hospital Bonn, 53127 Bonn, Germany; 43 Center for Behavioural Brain Sciences (CBBS), 39120 Magdeburg, Germany; 44 Biomedical Magnetic Resonance, Otto-von-Guericke University, 39120 Magdeburg, Germany

**Keywords:** Alzheimer’s disease, inferior frontal sulcal hyperintensity, glymphatic system, magnetic resonance imaging, fluid-attenuated inversion recovery, amyloid positivity

## Abstract

Inferior frontal sulcal hyperintensities (IFSHs) on fluid-attenuated inversion recovery (FLAIR) sequences have been proposed to be indicative of glymphatic dysfunction. Replication studies in large and diverse samples are nonetheless needed to confirm them as an imaging biomarker. We investigated whether IFSHs were tied to Alzheimer’s disease (AD) pathology and cognitive performance. We used data from 361 participants along the AD continuum, who were enrolled in the multicentre DELCODE study. The IFSHs were rated visually based on FLAIR magnetic resonance imaging. We performed ordinal regression to examine the relationship between the IFSHs and cerebrospinal fluid-derived amyloid positivity and tau positivity (Aβ42/40 ratio ≤ 0.08; pTau181 ≥ 73.65 pg/mL) and linear regression to examine the relationship between cognitive performance (i.e., Mini-Mental State Examination and global cognitive and domain-specific performance) and the IFSHs. We controlled the models for age, sex, years of education, and history of hypertension. The IFSH scores were higher in those participants with amyloid positivity (OR: 1.95, 95% CI: 1.05–3.59) but not tau positivity (OR: 1.12, 95% CI: 0.57–2.18). The IFSH scores were higher in older participants (OR: 1.05, 95% CI: 1.00–1.10) and lower in males compared to females (OR: 0.44, 95% CI: 0.26–0.76). We did not find sufficient evidence linking the IFSH scores with cognitive performance after correcting for demographics and AD biomarker positivity. IFSHs may reflect the aberrant accumulation of amyloid β beyond age.

## 1. Introduction

The cerebrospinal fluid (CSF) in the regions immediately superior to the cribriform plate, particularly those corresponding to the inferior frontal sulci, may display elevated signal intensities in T2-weighted fluid-attenuated inversion recovery (FLAIR) imaging [1]. The occurrence and extent of these hyperintensities, referred to as inferior frontal sulcal hyperintensities (IFSHs), have been proposed to be indicative of impaired CSF clearance and glymphatic dysfunction [2]. This is because CSF is presumed to enter the meningeal lymphatics in close proximity to the cribriform plate. The stagnation of proteins, blood, and cell debris in this region could lead to such hyperintensities [1].

To date, two studies have investigated IFSHs in humans. Zhang et al. [2] proposed the first visual rating scheme for assessing IFSHs and showed that IFSHs are more common in older participants as well as in those with more perivascular spaces—another presumed imaging marker of glymphatic dysfunction [3]. Due to a lack of amyloid and tau markers in those seminal studies, the connection between IFSHs and amyloid or tau pathology could not be established yet. In participants without manifest dementia, Xu et al. [4] sought to assess whether IFSHs reflected cerebral small vessel disease (CSVD) and amyloid or tau accumulation in brain parenchyma. Using amyloid and tau positron emission tomography (PET), the IFSHs were associated with CSVD but not with the PET-derived amyloid or tau concentrations. As the study was restricted to cognitively normal control (NC) subjects and subjects with mild cognitive impairment (MCI), this prevents more (disease-)specific assumptions yet and also raises the question of whether IFSHs might be a late consequence in the course of Alzheimer’s disease (AD). Those are the only previous studies exploring the potential role of IFSHs as non-invasive biomarkers of altered CSF clearance [2,4]. Therefore, further research in larger, more diverse samples is warranted, including patients on the AD continuum with available amyloid and tau data. Since AD is characterized by progressive neurodegeneration with the accumulation of waste proteins in the brain and glymphatic dysfunction, it might be linked to IFSHs [4]. 

Here, we assessed the IFSHs in a German multicentre study sample with participants on the AD continuum recruited from memory clinics. Our interests were two-fold. First, assuming that IFSHs reflect impaired glymphatic function, we aimed to explore the association between IFSHs and abnormal amyloid β (Aβ) accumulation or tau pathology [5], which could extend beyond the influence of age. Second, if IFSHs are indicative of the buildup of waste proteins and cellular debris, they should be related to cognitive consequences.

## 2. Materials and Methods

### 2.1. Study Design and Sample

In this study, we analysed data from the multicentre observational DZNE-Longitudinal Cognitive Impairment and Dementia Study (DELCODE) in Germany (for detailed information, see [6]). The study sample included AD dementia, MCI, and subjective cognitive decline (SCD) patients as well as NC subjects. Patient groups (AD dementia, MCI, and SCD) were referrals to the participating university-based memory clinics. NC participants were recruited by standardized public advertisement. Before enrolment in the study, all participants underwent a comprehensive assessment at the local study sites, including extensive clinical and neuropsychological testing (harmonized across all sites), magnetic resonance imaging (MRI), sampling of blood, urine, and CSF, and medical history. 

Participants with AD dementia, MCI, and SCD fulfilled the current research criteria [7,8,9]. To evaluate cognition, the Consortium to Establish a Registry for AD-plus (CERAD-plus) neuropsychological test battery was utilized at all memory clinics. To qualify for DELCODE, AD patients required a Mini-Mental State Examination (MMSE) score ≥ 18 points and a CERAD-plus neuropsychological test score below −1.5 standard deviations (SD) of the age-, sex-, and education-adjusted normal performance, MCI patients a score below −1.5 SD of the age-, sex-, and education-adjusted delayed recall trial of the CERAD-plus world-list episodic memory tests, and SCD patients a CERAD-plus neuropsychological test score better than −1.5 SD below the age-, sex-, and education-adjusted normal performance on all subtests. NC participants were required to perform within 1.5 SD in the CERAD-plus neuropsychological test. 

Further inclusion criteria for all participants were age ≥ 60 years, fluent German language skills, and the ability to provide informed consent. Exclusion criteria were current major depressive episode, major psychiatric disorders at baseline or in the past (e.g., psychotic disorder, bipolar disorder, or substance abuse), neurodegenerative disorders other than AD, vascular dementia, history of malignant disease or stroke with residual clinical symptoms, clinically significant abnormalities in vitamin B12, chronic use of psychoactive medication with sedative or anticholinergic effects, application of anti-dementia medication in MCI, SCD, and NC, and investigational medication for the treatment of dementia or cognitive impairment one month before entry and for the length of study. 

All participants provided written informed consent according to the Declaration of Helsinki. DELCODE was retrospectively registered at the German Clinical Trials Register (DRKS00007966, 4 May 2015). The local Ethics Committees of the participating sites (Berlin, Bonn, Cologne, Goettingen, Magdeburg, Munich, Rostock, and Tuebingen) approved the study.

### 2.2. Measurements

In addition to demographics, such as age, sex, years of education, and clinical diagnoses, we also considered further variables, including diagnosis of arterial hypertension (categorized into normotensive or hypertensive according to their International Statistical Classification of Diseases and Related Health Problems (ICD-10) [10]) and cognitive function.

Cognitive function was measured by the MMSE and the preclinical Alzheimer’s cognitive 5 (PACC5), which is sensitive to cognitive change in preclinical AD [11]. Moreover, global cognition and performance in five cognitive domains were assessed, which were derived from a confirmatory factor analysis on a comprehensive neuropsychological test battery [12]. The cognitive domains comprised learning and memory, executive function, language abilities, visuospatial functions, and working memory.

Amyloid positivity (A+) and phosphorylated tau (p-tau) positivity (T+) were determined by Aβ42/40 ratios and p-tau181 levels in CSF. We used DELCODE-specific cutoff values for A+ (≤0.08) and T+ (≥73.65 pg/mL) [13]. A+T+ was considered as AD pathology according to the National Institute on Aging-Alzheimer’s Association (NIA-AA; [14]). 

### 2.3. Brain MRI Acquisition and Processing

T2-weighted FLAIR sequences were acquired at nine scanning sites equipped with 3T Siemens MR tomographs (Siemens, Erlangen, Germany) using standardized DELCODE MR protocols [6], which included high-resolution FLAIR images (full head coverage; repetition time = 5000 ms; echo time = 394 ms; inversion time = 1800 ms; voxel size = 1 mm^3^ isotropic). The provision of standard operating procedures (SOP) for the implementation of each protocol and quality assurance were ensured by the DZNE imaging network. Every radiographer, who operated MRI scanners, received centralized training to implement the SOP [6]. 

### 2.4. Method of IFSHs Rating

**Figure 1:**
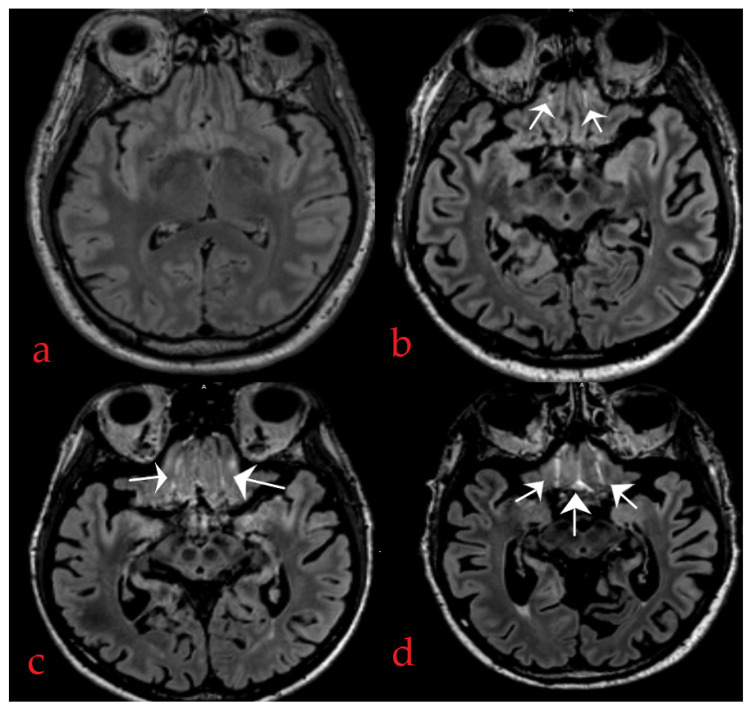
Example of inferior frontal sulcal hyperintensity (IFSH) on fluid-attenuated inversion recovery (FLAIR) images. The IFSH score in each of the three inferior frontal sulci was assessed above the reference slice. (**a**) The IFSH total score was 0. (**b**) The IFSH total score was 2 (see white arrows: 1 point for the right sulcus and 1 point for the left sulcus). (**c**) The IFSH total score was 5 (three points for the right sulcus and two points for the left sulcus). (**d**) The total IFSH score was 8 (three points for each the right and left sulcus, and two points for the central sulcus.

The IFSHs, defined as hyperintense CSF signals on FLAIR in one or more of the three inferior frontal sulci, encompassing the central sulcus between the gyri recti and both the right and left olfactory sulci, were rated according to the user guide by Lim et al. [15]. In a first step, all FLAIR sequences were orientated parallel to the floor of the anterior cranial fossa utilizing multi-planar reconstruction (MPR). Subsequently, the reference slice clearly displaying all three sulci was identified. Then, we analysed each sulcus above the reference slice over multiple axial slices and documented the maximum sulcal length affected. Each sulcus is scored separately with a score range of 0 to 3 points (0 = none of the sulcus affected; 1 = less than half of the sulcus length affected; 2 = at least half of the sulcus length affected; and 3 = most or whole of the sulcus length affected), adding all three sulci scores to a total range of 0 to 9 points (Figure 1). The total IFSH score was divided into three categories (0–1, 2–4, or 5–9) in accordance with the distribution of the total scores among our participants. This approach is similar to [4].

The IFSH scores were assessed by a trained rater, a resident in Neurology (M.D.), using Mango Software (version 4.1, 25 March 2019) [16]. MRI scans of *n* = 20 participants were chosen randomly and scored a second time at least four weeks after the initial MRI analyses by the same Neurology resident (M.D.), and also by another Neurology resident (K.S.). Both raters were trained and blinded to all clinical data. Overall, Cohen’s kappa demonstrated good intra- and inter-rater consistency for the right sulci (kappa_intra_ = 0.808, kappa_inter_ = 0.733), central sulci (kappa_intra_ = 0.815, kappa_inter_ = 0.706), and left sulci (kappa_intra_ = 0.811, kappa_inter_ = 0.643).

### 2.5. Statistical Analysis

We used IBM SPSS Statistics for Windows, Version 29 (Armonk, NY, USA: IBM Corp.), for statistical analysis. Patient characteristics were described by calculating mean and median scores, SD, and relative and absolute distributions. To compare normally distributed continuous variables, we applied a one-way ANOVA and chi-squared test to evaluate differences between normally distributed categorical variables. Collinearity statistics were utilized to identify issues of multicollinearity. We divided our regression models into potential (1) factors contributing to IFSHs and (2) outcomes associated with IFSHs. (1) In a first step, we performed univariate ordinal regression analyses with the total IFSH score (categories) as dependent variable and age, sex, years of education, and the dichotomous variables arterial hypertension, A+, T+, and AD pathology (A+ and T+ combined) as independent variables separately. Secondly, a multivariable ordinal regression analysis, which included all independent variables (except for AD pathology), was conducted. Due to the high correlation between A+, T+, and AD pathology, we re-performed the multivariable regression in a third step, excluding A+ and T+, and including AD pathology as independent variable. (2) The same procedure was repeated to determine the effect of the total IFSH score (categories) and the other variables from the first regression model on the different cognitive scores (see Section 2.2) using a linear regression model. Significance level was set at *p* < 0.05 (two-sided *p*-value) and adjusted for multiple comparisons by post-hoc Bonferroni correction and chi-squared testing. 

## 3. Results

### 3.1. Description of the Study Sample

We included 361 participants (Table 1; 46/79/156/80: AD dementia/MCI/SCD/NC; mean age 70.97 (SD 5.78) years; 175 women; total IFSH score 3.11 (SD 1.49) points; A+: 43.8%; T+: 24.7%; A+T+: 21.9%). The subjects with AD and MCI were older (*p* ≤ 0.001 and *p* = 0.024), had lower MMSE scores (*p* ≤ 0.001), and had more CSF biomarker positivity than the NC subjects (*p* ≤ 0.001 for A+, T+, and AD pathology). The total IFSH sum scores were higher in AD vs. MCI (3.63 vs. 2.87 points; *p* = 0.036). 

### 3.2. Associations between IFSHs and AD Pathology

Table 2 illustrates the associations between the total IFSH score (categories) as the dependent variable and demographic and clinical data as independent variables (potential factors contributing to IFSHs). There was no evidence of collinearity. Both the variance inflation factor (VIF) and tolerance for each independent variable were below and above, respectively, the values suggested in the literature (VIF < 2.5 and tolerance > 0.4; [17]). 

The multivariable regression models (Step 2) suggested that older age, female sex, and A+ were associated with higher IFSH scores. The odds of having higher total IFSH scores were 95% higher in the subjects with A+ than those with A− (odds ratios (OR): 1.95, 95% confidence interval (CI): 1.05 to 3.59, *p* = 0.032). The other independent variables did not reach statistical significance. Interestingly, A+ still predicted higher total IFSH scores after excluding the AD and MCI participants (OR: 2.44, 95% CI: 1.20 to 4.95, *p* = 0.014); see Supplementary Table S1). In the third step, only older age and female sex were significantly associated with higher IFSH scores, while AD pathology (A+ and T+ combined) did not reach statistical significance.

A one-way ANOVA comparing the total IFSH scores (categories) between the biomarker profiles (not A+ and not T+ defined as A−T−, A+ but not T+ defined as A+T−, A+T+, not A+ and T+ defined as A−T+) indicated significant differences on a group level (*p* = 0.003). The Bonferroni correction demonstrated significant differences between A−T− vs. A+T− (*p* = 0.029) and A−T− vs. A+T+ (*p* = 0.016) but not between A+T− vs. A+T+ or A−T− vs. A−T+.

A second regression analysis, exploring the potential cognitive repercussions of the IFSHs, was applied (see Supplementary Table S2). Therefore, we used different cognitive scores as dependent variables separately: the total MMSE score, global cognitive performance, domain-specific performance (learning and memory, executive function, language abilities, visuospatial functions, and working memory), and the PACC5 (see Section 2.2). We did not find evidence for a relationship between the total IFSH scores (categories) and cognitive function after controlling for age, sex, years of education, arterial hypertension, A+, T+, and AD pathology.

## 4. Discussion

We tested whether IFSHs were associated with AD pathology (A+ and T+) and cognitive performance in a sample of 361 participants across the AD continuum. We found that the IFSHs were associated with amyloid positivity but not tau positivity and that this relationship remained after correcting for age, sex, years of education, and a history of hypertension. However, we did not find sufficient evidence suggesting that the IFSHs were tied to cognitive performance beyond demographics, arterial hypertension, and AD biomarker positivity.

Our work indicated that higher IFSH scores were associated with increasing age, in agreement with previous works [2]. Past studies suggested that larger molecules, cell debris, or proteins could alter the CSF MRI signal intensity [18]. These metabolites of the brain are found more often with increasing age, e.g., due to brain atrophy, which is associated with the loss of neurons and glial cells [4,19,20]. Of note, we found IFSHs to occur in both atrophic and non-atrophic brains (see also [4]). Since the inferior frontal sulci, located superior to the cribriform plate, are a main location of glymphatic CSF clearance [18], the CSF hyperintensities on MRI scans are expected to occur in this region and particularly in older adults, who demonstrated decreased glymphatic function [19].

However, glymphatic dysfunction is not only an age-related phenomenon [21] but also plays a pivotal role in numerous pathologies, such as idiopathic normal pressure hydrocephalus or AD [22,23,24]. Apart from progressive neurodegeneration and glymphatic dysfunction, a hallmark of AD is Aβ deposition, as well as higher rates of neurotoxic waste production [25,26]. Interestingly, multivariable regression analyses suggested that IFSHs are associated with A+ beyond age. Additionally, recent findings indicated that Aβ is deposited in the orbitofrontal cortex, which is in close vicinity to IFSHs [5,27]. Our observed IFSH score differences between the biomarker profiles reinforce the hypothesis that A+ rather than T+ is needed for IFSHs to become visible on FLAIR images. Hence, IFSHs might reflect Aβ accumulation. Consequently, the increased deposition of Aβ caused by glymphatic dysfunction might lead to further ischemic and hypoxic reactions [28,29], aggravate the microglial inflammatory response, and ultimately foster inflammation and the progression of AD [30,31]. 

As a cautionary note, Xu et al. [4] did not find any associations between IFSHs and AD biomarkers. Still, their study was limited to NC and MCI subjects and only a small amount of PET-derived amyloid or tau data. The authors suggested that a lack of late-stage patients on the AD continuum might have restricted more disease-specific assumptions in their study: in the early stages of the disease, the pathological damage may be small. The inclusion of AD dementia patients in our study and the significant associations between A+ and the IFSH scores beyond age suggest that IFSHs could be a reflection of the long-term accumulation of Aβ rather than of the initial disease stages. Still, A+ predicted higher IFSH scores even after excluding AD dementia and MCI participants in our analyses. Future studies should strive to unravel the complex relationships between IFSHs and the AD continuum to determine whether IFSHs are potential early disease biomarkers or instead late-stage findings. 

Another finding of our study is the association between female sex and higher IFSH scores. This finding is in accordance with prior research indicating that women are two to three times more likely to develop AD than men [32]. There are multiple lifestyle factors known to promote the development of AD, which are particularly often found in women. For instance, women are especially vulnerable to sleep disruption around the transition to menopause, and sleep disturbances have been linked to an increase in Aβ [32,33]. Pregnancy might also affect the cerebrovascular system and thus glymphatic function in the long term [32]. 

Finally, higher IFSH scores did not result in cognitive repercussions. Our finding might be explained by the distribution of A+ and T+ in our study sample (A+ > T+), which is to be expected considering the pathophysiological mechanism of AD development, in which the brain initially accumulates Aβ followed by a p-tau pathology. A recent study identified the rate of p-tau changes to mediate the relationship between the initial amyloid manifestation and final cognition [34]. Thus, future studies might want to focus on the intertwined relations of A+, T+, and cognitive function in the context of IFSHs. 

There are some limitations to this study. First, our study is cross-sectional, and, therefore, further longitudinal studies are warranted to explore the causal relationships between IFSHs and other variables. Second, as of yet, it is not possible to obtain CSF samples from the inferior frontal sulci, and thus the definite nature of IFSHs remains unclear. Although our findings align with recent investigations [2,4] suggesting that IFSHs reflect glymphatic dysfunction, cerebral blood vessel leakage or tissue lesions due to aging might be other possible explanations. That is, blood, proteins, or cell debris might enter the brain instead of insufficient drainage from the brain leading to IFSHs. Future histopathological studies, sophisticated neuroimaging techniques (e.g., MR spectroscopy or magnetization transfer imaging), and more diverse study samples might elucidate this question. Moreover, the analysis of additional MRI sequences not restricted to FLAIR images might help to understand the potential cause of IFSHs. Certainly, the IFSH location poses a challenge due to a strong susceptibility to artefacts in this region. Hence, the existence of imaging artefacts cannot be ruled out. Those artefacts might depend on patients’ characteristics, such as head size, body mass index, or brain atrophy. Further MR-related aspects, such as imperfect inversion, might be another cause of such artefacts [35]. Still, we did not observe any artefacts in the IFSH regions aside from those already mentioned by [15] (see Supplementary Figure S1). 

## 5. Conclusions

Our study provides evidence of an association between IFSHs and amyloid pathology that persists even after accounting for demographics and vascular risk factors. IFSHs could therefore represent an atypical neuroimaging finding that could be widely assessed as a routine clinical procedure. Our findings should, however, be treated with caution given that the definite nature of IFSHs remains elusive. 

## Figures and Tables

**Table 1:** Sociodemographic and clinical characteristics of the study sample. Information regarding history of arterial hypertension was available for most but not all participants (354/361).

	Overall (*n* = 361)	AD Dementia (*n* = 46)	MCI (*n* = 79)	SCD (*n* = 156)	NC (*n* = 80)	*p*-Value (*p* < 0.05)
Age, y	70.97 (5.78)	74.78 (5.85)	71.56 (5.56)	70.55 (5.79)	69.0 (4.84)	**<0.001**
Male, *n* (%)	186 (51.50)	16 (34.8)	44 (55.7)	88 (56.4)	38 (47.5)	0.052
Years of education	14.35 (2.97)	13.11 (3.11)	13.73 (2.84)	14.98 (2.96)	14.43 (2.72)	**<0.001**
Arterial hypertension, *n* (%)	193 (54.51)	27 (58.7)	43 (56.57)	86 (56.57)	37 (46.3)	0.404
MMSE total score	28.13 (2.63)	23.02 (3.37)	27.80 (0.34)	29.15 (1.08)	29.40 (0.82)	**<0.001**
Aβ positivity, *n* (%)	158 (43.8)	41 (89.1)	47 (59.5)	52 (33.3)	18 (22.5)	**<0.001**
p-tau positivity, *n* (%)	89 (24.70)	32 (69.6)	30 (38.0)	22 (14.1)	5 (6.3)	**<0.001**
AD pathology (yes), *n* (%)	79 (21.90)	32 (69.6)	28 (35.40)	16 (10.3)	3 (3.8)	**<0.001**
IFSH score						
Right sulcus	1.10 (0.74)	1.35 (0.76)	1.08 (0.69)	1.08 (0.79)	1.02 (0.67)	0.106
Central sulcus	0.80 (0.53)	0.89 (0.43)	0.77 (0.45)	0.79 (0.60)	0.79 (0.54)	0.651
Left sulcus	1.21 (0.80)	1.39 (0.71)	1.04 (0.68)	1.28 (0.87)	1.14 (0.77)	**0.048**
Total IFSH sum score	3.11 (1.49)	3.63 (1.25)	2.87 (1.36)	3.15 (1.61)	2.95 (1.42)	**0.034**

Note: *n*: number. y: years. AD: Alzheimer’s disease. MCI: mild cognitive impairment. SCD: subjective cognitive decline. NC: cognitively normal control. MMSE: Mini-Mental State Examination. Aβ: amyloid β. p-tau: phosphorylated tau. IFSH: inferior frontal sulcal hyperintensity. Values are mean (standard deviation) unless otherwise noted. Significant *p*-values are marked bold.

**Table 2:** Associations between total IFSH score, demographics, and clinical data.

	Step 1 Univariate		Step 2 Multivariable		Step 3 Multivariable	
Variables	OR (95% CI)	*p*-Value	OR (95% CI)	*p*-Value	OR (95% CI)	*p*-Value
Age	1.07 (1.02 to 1.11)	**0.002**	1.05 (1.00 to 1.10)	**0.020**	1.07 (1.02 to 1.11)	**0.003**
Male sex	0.49 (0.30 to 0.80)	**0.005**	0.44 (0.26 to 0.76)	**0.004**	0.46 (0.27 to 0.80)	**0.006**
Years of education	0.91 (0.84 to 0.99)	**0.042**	0.97 (0.89 to 1.06)	0.641	0.96 (0.88 to 1.05)	0.471
Arterial hypertension	1.67 (1.02 to 2.74)	**0.041**	1.55 (0.94 to 2.57)	0.084	1.53 (0.93 to 2.53)	0.093
Aβ positivity	2.33 (1.41 to 3.86)	**<0.001**	1.95 (1.05 to 3.59)	**0.032**		
p-tau positivity	1.99 (1.14 to 3.46)	**0.014**	1.12 (0.57 to 2.18)	0.727		
AD pathology	1.84 (1.04 to 3.28)	**0.035**			1.40 (0.76 to 2.59)	0.276

Note: OR: odds ratio. CI: confidence interval. Step 1: univariate regression model. Step 2: multivariable regression model, including age, sex, years of education, arterial hypertension, Aβ positivity, and p-tau positivity as independent variables. Step 3: multivariable regression model, including age, sex, years of education, arterial hypertension, and AD pathology as independent variables. Significant *p*-values are marked bold.

## Data Availability

The data presented in this study are available on request from the corresponding author.

## Supplementary Materials

The following supporting information can be downloaded at https://www.mdpi.com/article/10.3390/diagnostics14090940/s1, Table S1: Associations between total IFSH score, demographics and clinical data—NC and SCD participants only. Table S2: Associations between total IFSH score and cognitive scores. Figure S1: Example of Inferior Frontal Sulcal Hyperintensity (IFSH) on fluid-attenuated inversion recovery (FLAIR) images in different planes and artefacts in the middle sulcus.
