# Supplementary material for: Inferior Frontal Sulcal Hyperintensities on Brain MRI Are Associated with Amyloid Positivity beyond Age—Results from the Multicentre Observational DELCODE Study
Source: Diagnostics (Basel). 2024 Apr 30;14(9):940. doi: 10.3390/diagnostics14090940 (PMC11083612; doi:10.3390/diagnostics14090940)
Supplement: Supplementary file 1 [file diagnostics-14-00940-s001.zip › diagnostics-2962143-supplementary.pdf]

Supplementary Table S1. Associations between total IFSH score, demographics and clinical data—NC and SCD participants only.

	Step 1 Multivariable		Step 2 Multivariable	
**Variables**	**OR (95% CI)**	**p-value**	**OR (95% CI)**	**p-value**
Age	1.05 (0.99 to 1.11)	0.067	1.06 (1.00 to 1.12)	**0.028**
Male sex	0.33 (0.17 to 0.65)	**0.001**	0.37 (0.19 to 0.72)	**0.004**
Years of education	0.90 (0.88 to 1.11)	0.857	0.83 (0.88 to 1.09)	0.749
Arterial hypertension	1.73 (0.52 to 3.20)	0.079	1.73 (0.93 to 3.19)	0.078
Aβ positivity	2.44 (1.20 to 4.95)	**0.014**		
p-tau positivity	1.70 (0.64 to 4.46)	0.278		
AD pathology			2.69 (0.91 to 7.04)	0.072

Note: IFSH: Inferior Frontal Sulcal Hyperintensity (IFSH) score (categories) as dependent variable. NC: Cognitively normal control. SCD: subjective cognitive decline. OR: odds ratio. CI: confidence interval. Step 1: multivariable regression model, including age, sex, years of education, arterial hypertension, Aβ positivity, and p-tau positivity as independent variables. Step 2: multivariable regression model, including age, sex, years of education, arterial hypertension, and AD pathology as independent variables. Significant p-values are marked bold.

Supplementary Table S2. Associations between total IFSH score and cognitive scores.

	Step 1 Univariate	Step 2 Multivariable	Step 3 Multivariable
**Cognitive scores[a]**	**β (95% CI)**	**β (95% CI)**	**β (95% CI)**
Mini-Mental State Examination	-0.25 (-0.81 to 0.29)	0.33 (-0.16 to 0.84)	0.21 (-0.27 to 0.71)
Global cognitive performance	-10.62 (-34.95 to 13.70)	11.54 (-11.19 to 34.28)	8.22 (-14.26 to 30.72)
Learning and memory	-8.08 (-32.72 to 16.55)	8.18 (-14.77 to 31.14)	4.82 (-17.79 to 27.44)
Executive function	-7.84 (-31.97 to 16.29)	14.99 (-7.77 to 37.76)	12.59 (-9.95 to 35.14)
Language abilities	-8.19 (-32.54 to 16.15)	11.08 (-11.97 to 34.13)	7.52 (-15.29 to 30.35)
Visuospatial functions	-21.99 (-46.18 to 2.19)	-4.16 (-28.07 to 19.75)	-5.87 (-29.56 to 17.82)
Working memory	**-27.11 (-51.16 to -3.07)**	-11.64 (-35.34 to 12.06)	-11.06 (-34.54 to 12.41)

Preclinical Alzheimer's cognitive composite 5	-0.46 (-15.36 to 14.43)	-7.29 (-22.60 to 8.01)	-6.41 (-21.62 to 8.79)

Note: CI: confidence interval. Step 1: univariate regression model. Step 2: multivariable regression model, including age, sex, years of education, arterial hypertension, Aβ positivity, phosphorylated tau (p-tau) positivity and the total Inferior Frontal Sulcal Hyperintensity (IFSH) score (categories) as independent variables. Step 3: multivariable regression model, including age, sex, years of education, arterial hypertension, Alzheimer's disease (AD) pathology and the total IFSH score as independent variables. Dependent variables: [a] Results are shown for independent variable total IFSH score. Significant p-values are marked bold.

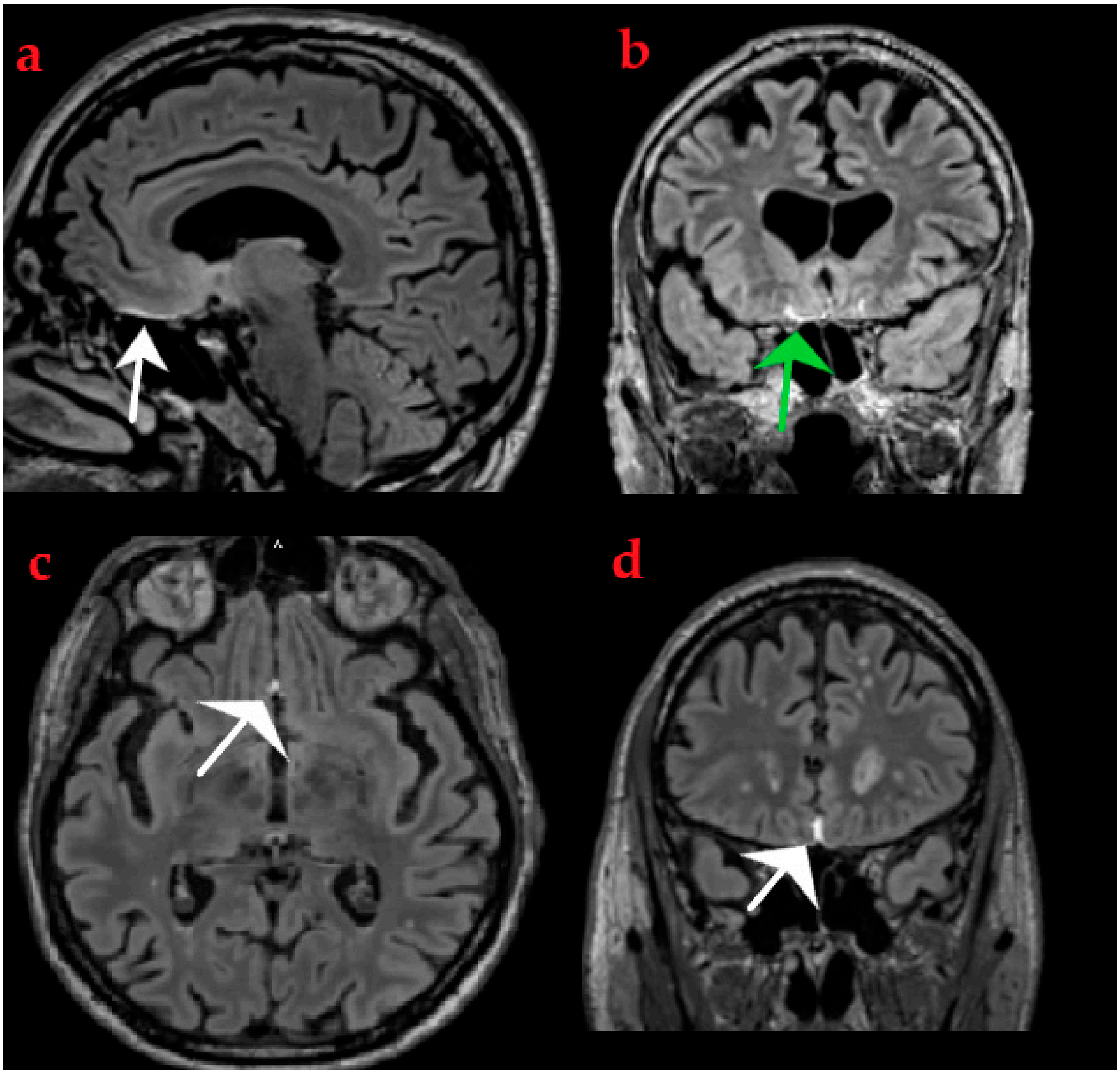

Supplementary Figure S1. Example of Inferior Frontal Sulcal Hyperintensity (IFSH) on fluid-attenuated inversion recovery (FLAIR) images in different planes and artefacts in the middle sulcus. a: sagittal plane; white arrow points towards IFSH. b: coronal plane; green arrow points towards IFSH. c and d: white arrows point towards single focus of hyperintensity in the posterior aspect of the middle

sulcus. Those hyperintensities are related to the presence of blood vessels or the optic chiasm and were not considered for the scoring of IFSH. Note: IFSH-related artefacts in a and b would have spherical appearance, which is not the case.
